# Functional and Morphological Plasticity of the Endolysosomal System: Pigment Organelles at the Crossroads of Physiology and Pathology

**DOI:** 10.1111/boc.70036

**Published:** 2025-10-03

**Authors:** Laura Salavessa, Myckaëla Rouabah, Paula Pernea, Smail Hadj‐Rabia, Cédric Delevoye

**Affiliations:** ^1^ Université Paris Cité, INSERM UMR‐S1151, CNRS UMR‐S8253 Institut Necker Enfants Malades Paris France; ^2^ Department of Dermatology Hôpital Necker‐Enfants Malades, APHP‐Centre Paris France; ^3^ Reference Center for Rare Skin Diseases (MAGEC Nord‐Necker) Filière FIMARAD, ERN‐skin, Hôpital Necker‐Enfants Malades, APHP‐Centre Paris France; ^4^ Université Paris Cité, INSERM U1163 Institut Imagine Paris France

**Keywords:** endolysosomal system, lysosome‐related organelles, membrane dynamics, skin diseases, skin pigmentation and photoprotection

## Abstract

The endolysosomal system is a highly dynamic and versatile network of organelles essential for maintaining cellular and tissue homeostasis. Its functional diversity relies on a high degree of plasticity, driven by tightly regulated membrane remodeling and intracellular trafficking events. In certain specialized cells, this plasticity enables the formation of lysosome‐related organelles, like melanosomes in pigment cells, through the repurposing of ubiquitous membrane trafficking machineries. Disruption of these pathways can lead to pathological conditions, including genetic disorders. In this review, we explore how endolysosomal plasticity underlies key adaptive cellular strategies at the cellular and tissue levels. Focusing on melanocytes, which synthesize melanin, and keratinocytes, which receive and store it, we illustrate how trafficking and membrane dynamics events coordinate between these two cell types for skin pigmentation and photoprotection, and how mutations affecting these processes lead to genetic forms of albinism. By using skin pigmentation as a model of cell‐ and tissue‐specific adaptation, this review highlights the broader physiological and pathological implications of endolysosomal membrane morphodynamics.

## Graphical abstract

The morphodynamical plasticity of the endolysosomal system supports the formation and function of specialized intracellular compartments, as exemplified by the pigment organelles in skin cells. In epidermal melanocytes and keratinocytes, membrane trafficking and remodeling coordinate tissue pigmentation and protect the genome from photodamage. Disruptions in these processes underlie genetic disorders such as certain forms of albinism, offering broader insights into endolysosomal system dynamics in health and disease.

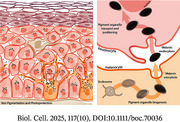

## 1 Introduction

Eukaryotic cells are distinguished by their membrane‐bound compartments, which create specialized environments for biochemical reactions. Consequently, the exchange of material between organelles relies, at least in part, on tubulovesicular trafficking—a process driven by the local remodeling of the organelle's limiting membrane. This remodeling allows the formation, transport, and delivery of cargo‐loaded carriers (e.g., proteins and lipids) from a source organelle to a target membrane. Among these trafficking pathways, endocytosis, exocytosis, and recycling routes act at the interface between the cell's exterior and interior, coordinating cargo transport as well as organelle biogenesis, maturation, and function.

At the core of these intracellular trafficking routes lies the endolysosomal system, a network of morphodynamic organelles crucial for cellular adaptation to environmental cues by performing both canonical functions across all cell types, and specialized functions in specific cell types (Da Graça et al. 2025). Early/Sorting endosomes serve as a cargo‐sorting station, directing material to the plasma membrane for recycling, or toward degradation via late endosomes/multivesicular bodies and lysosomes, which fuse to form degradative endolysosomes (Da Graça et al. 2025). Carriers’ formation in this system follows a series of membrane remodeling steps, such as the generation of a membrane hotspot where local curvature is induced, stabilized, and then elongated before fission of the membrane at its base to release the tubulo‐vesicle and allow for its transport. These processes are orchestrated by key molecular players, including phosphoinositides and adaptor protein complexes for cargo selection and concentration, curvature‐sensing proteins, cytoskeletal elements and molecular motors for membrane shaping and transport, and RAB GTPases for trafficking coordination. For example, budding structures emerging from the limiting membrane of early/sorting endosomes give rise to recycling endosomes, a tubulovesicular network that is continuously formed and consumed, and historically linked to receptor recycling to the plasma membrane (Geuze et al. 1983; Willingham et al. 1984). In addition, recycling endosomes contribute to various processes such as cell polarity, migration, division, phagocytosis of large particles, and organelle biogenesis (e.g., autophagosome, cilium, or lysosome‐related organelle [LRO]) (Cullen and Steinberg 2018).

The functional plasticity of the endolysosomal system is well illustrated in cells generating LROs—organelles formed in specialized cells and dedicated to the synthesis, storage, and secretion of their contents upon physiological stimulation (Delevoye et al. 2019). LROs represent a unique yet highly informative model system for studying endolysosomal dynamics. While their morphology, composition, and functions are tailored to specific cell types (e.g., melanosome in pigment cells, Weibel–Palade bodies (WPBs) in endothelial cells, dense granules in platelets, or lamellar bodies in lung alveolar type II epithelial cells), they largely rely on conserved membrane trafficking machinery for their biogenesis and function (Bowman et al. 2019; Delevoye et al. 2019). Notably, LROs are also termed endolysosome‐related organelles (ELROs) as they share features with lysosomes and most of their components derive from the endolysosomal system (Delevoye et al. 2019). Thus, LRO‐generating cells merge the peculiarities of specialized organelles with general principles of membrane dynamics, providing a powerful and physiologically relevant paradigm.

The melanosome, a prototypical LRO, exemplifies the dynamic interplay between endolysosomal trafficking and specialized organelle function. This melanin‐containing organelle, formed, for example, in skin epidermal melanocytes, originates from early/sorting endosomes and acquires most of its components from the recycling endosomal system (Le et al. 2021), while also intersecting the post‐Golgi secretory pathway (Patwardhan et al. 2017). Melanosome biogenesis and maturation involve specialized membrane remodeling events, culminating in melanin transport and transfer to neighboring keratinocytes (Le et al. 2021). In keratinocytes, transferred melanin is captured, internalized, and stored within other lysosome‐like pigment organelles (Benito‐Martínez et al. 2021; Correia et al. 2018; Hurbain et al. 2018), which may belong to the LRO family (Delevoye et al. 2019). These newly formed melanin organelles are strategically positioned perinuclearly and atop keratinocyte's nucleus, forming a “microparasol” to presumably shield their genome from UV‐induced DNA damage (Kobayashi et al. 1998). Thus, melanin is a photoprotective pigment produced in melanocytes and stored in neighboring keratinocytes of the epidermis, the skin's outermost layer that protects the organism against environmental aggressions, including UV solar radiation. This process relies on the tightly coordinated partnership between these two cells forming the epidermal melanin unit and involves two distinct organelles of the endolysosomal lineage. While melanosome biogenesis in melanocytes is relatively well‐studied, the membrane dynamics governing melanin storage and organization in keratinocytes remain comparatively poorly understood.

While the sequential process of melanin production, transfer, and storage is essential for establishing and maintaining normal pigmentation and photoprotection, defects in these pathways underlie a range of pigmentary disorders. Among them, albinism refers to a group of rare genetic disorders (global prevalence 1/17,000) primarily characterized by altered pigmentation and visual impairment (Mártinez‐García and Montoliu 2013). Pigmentation changes involve varying degrees of diffuse cutaneous and hair hypopigmentation, while the visual defects include ocular features as nystagmus, chiasmatic misrouting of the optic nerves, and foveal hypoplasia, resulting in moderate to severe reductions in visual acuity and pronounced photophobia linked to iris and fundus hypopigmentation (Mártinez‐García and Montoliu 2013). Albinism encompasses different subtypes, categorized into nonsyndromic forms, known as oculocutaneous albinism (OCA), and syndromic forms. In OCA, the primary defect lies in impaired melanin synthesis resulting in clinical manifestations restricted to skin and ocular features, and typically caused by mutations in genes encoding proteins essential for melanin production (e.g., melanin‐synthesizing enzymes TYR, TYRP1, TYRP2/DCT) (Grønskov et al. 2007). In syndromic subtypes—for example, the Hermanksy–Pudlak syndrome [HPS; global prevalence 1/250,000 (Lasseaux et al. 2018)]—albinism stems from defects in melanosome formation and/or function (Bowman et al. 2019; Wei, 2006). HPS arises from mutations in ubiquitously expressed genes encoding subunits of protein complexes with roles in membrane trafficking and LRO biology (Huizing et al. 2008) (MIM #203300; https://www.hpsnetwork.org). These complexes comprise the biogenesis of LRO complexes (BLOC‐1, BLOC‐2, BLOC‐3) and the adaptor protein complex AP‐3 (Bowman et al. 2019), which together underlie the 11 known HPS subtypes in humans (Li et al. 2022). In addition to the classical manifestations of albinism (i.e., hair and skin hypopigmentation and visual impairment), HPS phenotype also includes bleeding (e.g., epistaxis, bruising) attributed to the malformation and/or malfunction of dense granules—the LRO in platelets (Wei 2006)—and additional symptoms, such as granulomatous colitis, pulmonary involvement, or neurological symptoms in some subtypes.

HPS can therefore be defined as a membrane trafficking disorder that specifically affects LROs, as the melanosome, providing an ideal framework to dissect the membrane dynamics underlying organelle biology in skin pigmentation and photoprotection in health and disease. This review focuses on the membrane morphodynamic events and components known to be disrupted in HPS, with an emphasis on melanosome formation, maturation, and function in melanocytes, as well as a discussion on the underappreciated contribution of the endolysosomal system for pigment organelle biology in epidermal keratinocytes in the context of cutaneous photoprotection.

## 2 The Melanosome—the Pigment Factory

Melanin pigments are synthesized by melanocytes within melanosomes, a LRO that originates from the early endosomal system. Pigmented melanocytes produce hundreds of these melanin‐producing organellar factories, presenting a significant membrane challenge that necessitates a profound reorganization of their endomembrane system in favor of this specialized LRO. As a result, melanocytes have evolved an exceptionally specialized endolysosomal system—one that not only highlights its remarkable plasticity in specialized cell types, but also uncovers fundamental membrane dynamics events shared across virtually all cell types and disrupted in disease.

### 2.1 Endolysosomal Morphodynamics During Melanosome Biogenesis

**FIGURE 1:**
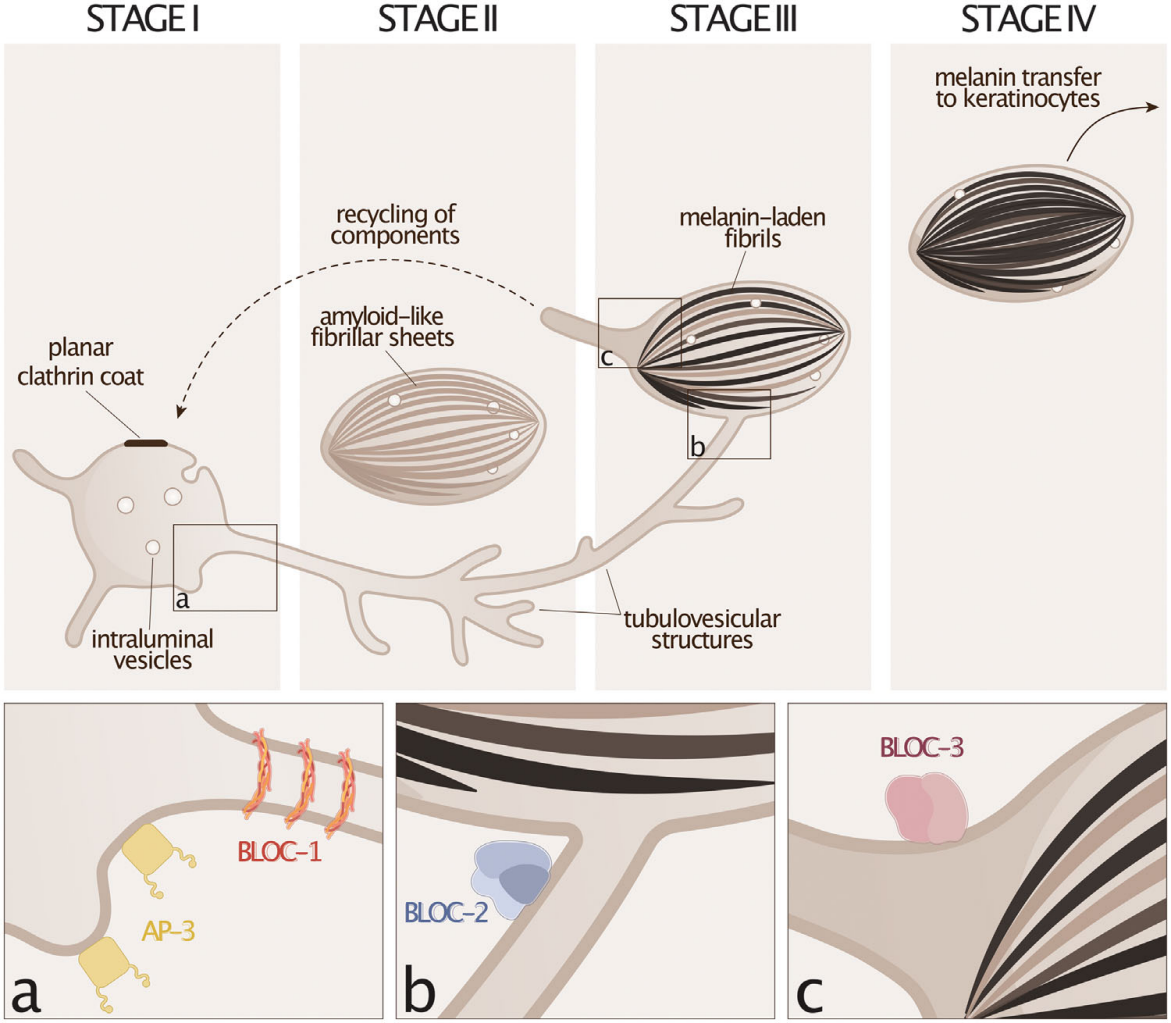
Membrane morphodynamics during melanosome biogenesis. Melanosomes develop in melanocytes from early/sorting endosomes through four distinct stages. Stage I melanosomes are early/sorting endosomes characterized by a vacuolar domain containing intraluminal vesicles and a planar clathrin coat, and a tubulovesicular network budding from the limiting membrane. The formation and elongation of intraluminal amyloid‐like fibrils reshape Stage I melanosomes into the distinctive ovoidal Stage II melanosomes. Both Stages I and II melanosomes are nonpigmented. Melanin‐laden fibrils become evident in Stage III melanosomes, which can be physically connected to Stage I melanosomes by tubular recycling endosomal structures emerging from the latter, thus facilitating the delivery of melanogenic cargoes. Fully pigmented Stage IV melanosomes are ultimately transferred to neighboring keratinocytes. (a) The octameric BLOC‐1 complex, comprising the subunits BLOC1S1 (BLOS1), BLOC1S2 (BLOS2), BLOC1S3 (BLOS3; HPS8), BLOC1S4 (cappuccino), BLOC1S5 (muted, HPS11), BLOC1S6 (pallidin; HPS9), BLOC1S7 (snapin), and BLOC1S8 (dysbindin; HPS7), forms a flexible, arc‐shaped rod that adapts to various membrane curvatures, contributing to the generation, elongation, and stabilization of endosomal‐derived membrane tubules arising from Stage I melanosomes. The adaptor complex AP‐3 regulates the selection of melanosomal cargoes such as TYR and promotes their packaging and transport into budding vesicles. (b) BLOC‐2, a trimeric complex comprised of HPS3, HPS5, and HPS6 subunits, associates with endosomal tubules in proximity to LROs. In melanocytes, BLOC‐2 aids in guiding endosomal‐derived tubules to Stage III melanosomes to enable cargo delivery. (c) BLOC‐3, a dimeric complex composed of HPS1 and HPS4 subunits, acts as a GEF for RAB32/RAB38 and would promote the formation of large membrane tubules from Stage III melanosomes that may recycle components back to early endosomes/Stage I melanosomes, supporting additional rounds of cargo delivery via the BLOC‐1‐dependent route.

Among LROs, melanosomes uniquely formed in pigment cells are well‐studied for their membrane dynamics processes during organelle formation and function. Initially described by biochemical and ultrastructural analysis (Baker et al. 1960; Drochmans, 1960; Seiji et al. 1961), melanosomes develop through four distinct maturation stages—Stages I/II are unpigmented, while Stages III/IV are pigmented. In melanocytes, Stage I melanosomes consist of conventional early/sorting endosomes, reinforcing their classification as LROs derived from the endolysosomal system. These early‐stage melanosomes, typically a few hundred nanometers in diameter, exhibit two morphologically distinct domains as follows: a vacuolar domain with an electron‐dense planar clathrin coat containing a few intraluminal vesicles, and nascent recycling endosomal tubulovesicular structures budding from its limiting membrane (Raposo et al. 2001) (Figure 1).

During melanosome biogenesis, the vacuolar domain of Stage I melanosomes evolves into the ovoidal Stage II through the formation and elongation of intraluminal amyloid‐like fibrillar sheets [not discussed here, readers can refer to original studies for details; Hurbain et al. 2008; Raposo et al. 2001; Rochin et al. 2013; van Niel et al. 2015, 2011] (Figure 1). Simultaneously, tubular structures emerging from the limiting membrane of Stage I melanosomes have been shown to form “membrane conduits” physically connected to some Stage III melanosomes (Delevoye et al. 2009) (Figure 1). This extensive endosomal membrane reshaping would enable the establishment of transient atypical membranous and luminal continuities between subpopulations of early endosomes and maturing LROs, and in particular between some Stage I and III melanosomes, thus facilitating the transport and delivery of cargoes required for melanin synthesis (Delevoye et al. 2016, 2009; Dennis et al. 2015; Di Pietro et al. 2006; Le et al. 2020; Mahanty et al. 2016; Setty et al. 2008; Sitaram et al. 2012; Zhu et al. 2022) (Figure 1). However, the mechanism governing cargo selection within the tubule—distinguishing components targeted to melanosomes from those that are not [e.g., TYRP1 vs. transferrin, respectively; Delevoye et al. 2009]—remains unknown. Finally, once melanogenesis initiates and melanin starts to deposit onto fibrillar sheets, Stage III melanosomes typically display electron‐dense, melanin‐laden fibrils that become further hidden by abundant intraluminal melanin in Stage IV melanosomes, which ultimately transfer melanin to keratinocytes (Le et al. 2021) (Figure 1, and see sections below).

### 2.2 Biogenesis and Membrane Targeting of Tubular Recycling Endosomes

The characterization of genes mutated in HPS has led to the identification of BLOCs as key regulators of LRO biology. Three BLOCs have been identified (BLOC‐1, BLOC‐2, BLOC‐3; see Table 1), each as a multisubunit protein complex with no shared components or functional overlap. From an evolutionary perspective, recent studies propose that precursors of all three BLOCs are found across diverse eukaryotic lineages and were likely present in the Last Eukaryotic Common Ancestor (LECA) (More et al. 2024). These ancestral complexes may have contributed to establish an ancestral endolysosomal system and endosome‐to‐lysosome membrane trafficking pathways (Cheli and Dell'Angelica 2010; More et al. 2024; Thomason et al. 2024). Consequently, and in line with the organelle paralogy hypothesis (Mast et al. 2014), the precursors of BLOCs were likely instrumental in the evolution of the eukaryote endomembrane system by enabling the emergence of novel sorting and trafficking pathways, thus contributing to the diversity of organelles seen in modern eukaryotes (More et al. 2024).

**TABLE 1:** Hermansky–Pudlak syndrome classification and characteristics.

Gene	Protein name	Complex	Function	LROs	HPS type	Clinical manifestation
*AP3B1*	AP‐3 complex subunit beta‐1	AP‐3	Four‐subunit complex involved in TYR sorting into endosome‐derived clathrin‐coated vesicles and some cargo transport via BLOC‐1‐dependent endosomal tubules to melanosomes	Melanosomes Platelet dense granules	HPS2	Hypopigmentation Ocular symptoms Bleeding diathesis Severe immunodeficiency: Neutropenia Impaired NK cytotoxicity Pulmonary fibrosis Neurological symptoms: Seizures Neurodevelopmental delays Impaired hearing
*AP3D1*	AP‐3 complex subunit delta‐1			Melanosomes Platelet dense granules (putatively) Neurotransmitter vesicles (putatively)	HPS10	
*BLOC1S3 (BLOS3/reduced pigmentation)*	BLOC‐1 subunit 3	BLOC‐1	Eight‐subunit complex involved in cargo transport from early endosomes to maturing melanosomes through the membrane‐tubulation process	Melanosomes Platelet dense granules	HPS8	Mild hypopigmentation Ocular symptoms Bleeding diathesis
*BLOC1S5 (muted)*	BLOC‐1 subunit 5			Melanosomes Platelet dense granules	HPS11	Mild hypopigmentation Ocular manifestation Bleeding diathesis
*BLOC1S6 (pallidin)*	BLOC‐1 subunit 6			Melanosomes Platelet dense granules Neurotransmitter vesicles	HPS9	Mild hypopigmentation Ocular manifestation Bleeding diathesis Reported cases of: Immunodeficiency Thrombocytopenia Leukopenia Schizophrenia Abnormal electroencephalogram
*BLOC1S8 (dysbindin)*	BLOC‐1 subunit 8			Melanosomes Platelet dense granules Neurotransmitter vesicles	HPS7	Mild hypopigmentation Ocular manifestation Bleeding diathesis Reported cases of: Granulomatous colitis Immunodeficiency
*HPS3*	HPS3	BLOC‐2	Three‐subunit complex facilitating BLOC‐1‐dependent cargo transport from endosomes to maturing melanosomes	Melanosomes Platelet dense granules	HPS3	Mild hypopigmentation Ocular symptoms Mild bleeding diathesis Granulomatous colitis
*HPS5*	HPS5			Melanosomes Platelet dense granules	HPS5	
*HPS6*	HPS6			Melanosomes Platelet dense granules	HPS6	
*HPS1*	HPS1	BLOC‐3	Two‐subunit complex promoting RAB32/RAB38 activation to facilitate recycling from pigmented melanosomes	Melanosomes Platelet dense granules	HPS1	Severe hypopigmentation Severe ocular symptoms Severe bleeding diathesis Lethal pulmonary fibrosis Granulomatous colitis
*HPS4*	HPS4			Melanosomes Platelet dense granules	HPS4	

#### 2.2.1 A Membrane Tubulating Machinery: A Role of BLOC‐1

BLOC‐1 is a hetero‐octameric protein complex, peripherally bound to membranes, and essential for the biogenesis of recycling endosomes in virtually all cell types (Delevoye et al. 2016). This pathway relies on the generation, elongation, and stabilization of membrane tubules arising from sorting endosomes, and that can ultimately establish physical connections with Stage III melanosomes in skin melanocytes (Delevoye et al. 2016, 2009) (Figure 1a). Mutations in four of the eight subunits composing BLOC‐1—BLOC1S8 (dysbindin), BLOC1S3 (BLOS3), BLOC1S6 (pallidin), and BLOC1S5 (muted)—are responsible for human HPS7, HPS8, HPS9, and HPS11 subtypes, respectively (Table 1). These HPS subtypes are primarily associated with albinism and bleeding diathesis, but have also been variably linked, depending on the subtype, to granulomatous colitis, immunodeficiency, thrombocytopenia, leukopenia, or neurological symptoms (Badolato et al. 2012; Boeckelmann et al. 2021; Liu et al. 2021; Lowe et al. 2013; Michaud et al. 2021; Okamura et al. 2018; Pennamen et al. 2020; Yousaf et al. 2016) (Table 1).

**FIGURE 2:**
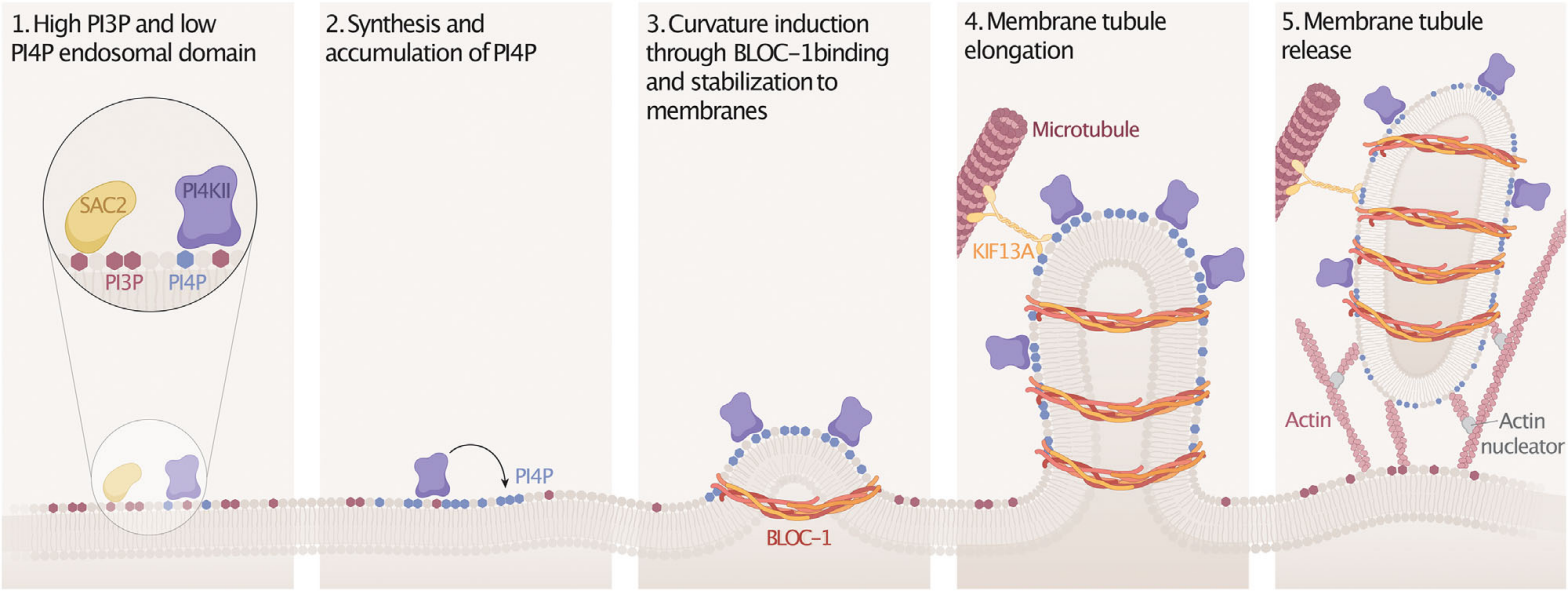
BLOC‐1 function in recycling endosomal membrane tubulation. At the limiting membrane of early endosomes, PI4P is locally synthesized by PI4KIIs at PI3P‐enriched subdomains and is likely rapidly depleted by SAC2 phosphatase activity (1). Local accumulation of PI4P would occur through sustained PI4KII activity (2), with the resulting PI4P‐rich subdomains promoting the recruitment, stabilization, and accumulation of BLOC‐1 on endosomal membranes. BLOC‐1 recruitment facilitates membrane curvature induction and tubule formation (3). As tubules elongate, they retain PI4P, which sustains continued BLOC‐1 enrichment and, together with KIF13A motors moving along microtubules, this exerts pulling forces that contribute to membrane tubule extension (4). Nascent recycling endosomal tubules are ultimately severed through the coordinated action of BLOC‐1 and actin polymerization machinery (5), releasing PI4P‐containing tubules essential for endosomal cargo recycling. Adapted from Jani et al. (2022).

Structurally, BLOC‐1 was recently proposed to exist as two distinct hemi‐complexes (i.e., BLOC1S2/5/7/8 and BLOCS1/3/4/6) that can merge to form a 42‐nm‐long flexible rod adopting an arc‐shaped architecture proposed to adapt to various membrane curvatures (Lee et al. 2012; Y. Wang et al. 2025), making it a strong candidate for local membrane remodeling during membrane tubulogenesis. In vitro, BLOC‐1 initiates membrane tubulation by preferentially binding to and accumulating on highly curved membranes enriched in negatively charged lipids (Jani et al. 2022) (Figure 2). This supports that BLOC‐1's primary function is to drive the transformation of membrane vesicles into tubules.

A critical factor in the initiation of this membrane tubulation process is the phosphatidylinositol‐4‐phosphate (PI4P), a phospholipid locally produced by PI4KIIα/β kinases—especially at PI3P‐rich early endosomal membranes (Zhu et al. 2022) (Figure 2). These PI4P‐rich endosomal subdomains would represent a “hot‐spot” facilitating BLOC‐1 membrane recruitment, stabilization, and accumulation, thus enabling membrane remodeling (Jani et al. 2022) (Figure 2). As a consequence, decreasing the expression of PI4KIIα/β is sufficient to abolish the initial membrane budding at early endosomes (Jani et al. 2022).

Other machineries must cooperate in generating/stabilizing the initial early endosomal membrane curvature, such as the AP‐1 complex that binds to PI4P‐rich membranes (Wang et al. 2003). In pigment cells as well as in other cell types, AP‐1 recruits and concentrates transmembrane cargos [e.g., TYRP1, ATP7A; Le et al. 2021] that can aid in locally remodeling membranes to create curvature signatures recognized and stabilized by BLOC‐1. AP‐1 is required for the endosome‐to‐melanosome trafficking pathway and interacts with KIF13A (Delevoye et al. 2009; Nakagawa et al. 2000), a plus‐end directed microtubule‐based kinesin motor and a RAB11 effector required for recycling endosome tubule formation and transport in nonpigment cells (Delevoye et al. 2014), and which can also complex with BLOC‐1 (Delevoye et al. 2016). Consequently, BLOC‐1 works in concert with lipid kinases, sorting adaptors, Rab GTPases, and molecular motors to not only elongate recycling tubules but also selectively enrich them with membrane‐associated cargos (e.g., TYRP1), before their strategic positioning near target organelles (Delevoye et al. 2009) (Figures 1 and 2). Finally, local branched actin polymerization and associated machineries (e.g., annexin A2, ARP2/3) further stabilize the elongated recycling tubules (Delevoye et al. 2016) and may ultimately contribute to their scission prior to their intracellular transport (Figure 2).

Beyond its role in endosomal membrane remodeling, BLOC‐1 is also involved in SNARE (Soluble NSF Attachment Protein Receptor)‐mediated membrane fusion, though its precise function remains unclear. BLOC1S6 or BLOC1S7 (a.k.a., snapin, BORCS3) can interact with syntaxin 13, a SNARE associated with recycling endosomes and required for melanosomal cargo delivery (Jani et al. 2015; Moriyama and Bonifacino 2002), or with plasma membrane SNAP25, respectively (Ilardi et al. 1999). Additionally, BLOC‐1 regulates the localization of VAMP7 (Salazar et al. 2006), a SNARE required for the fusion of recycling endosomal tubules with melanosomes, through a BLOC‐1/AP‐3 “super‐complex” [Bowman et al. 2021; see below].

BLOC‐1 is ubiquitously expressed alongside associated machineries such as AP‐1, KIF13A, and PI4KIIα/β. Given its expected conservation during eukaryote evolution, BLOC‐1 is likely a fundamental component within the modern endolysosomal system. Thus, and perhaps not surprisingly, BLOC‐1 enables endosomal tubulation across various cell types for diverse processes requiring recycling endosomes, including cargo transport to the plasma membrane [e.g., LDLR, TfR; Delevoye et al. 2016; Setty et al. 2007; Zhang et al. 2020] or the cilium (Monis et al. 2017), synaptic vesicle biogenesis (Chen et al. 2017), viral transport or bacterial niche establishment (Jani et al. 2022). Altogether, BLOC‐1 emerges as a central ancestral orchestrator of endosomal dynamics, coordinating the spatiotemporal remodeling of membranes into tubules enriched with specific cargos as they elongate, traffic, and fuse with target membranes, such as melanosomes for pigmentation.

#### 2.2.2 Endosomal GPS for Targeting LROs: A Role of BLOC‐2

Once BLOC‐1‐dependent recycling tubules are formed, they are targeted to specific membranes such as those of maturing melanosomes in pigment cells. This targeting relies on the expression of BLOC‐2 (Dennis et al. 2015), a hetero‐trimeric complex comprising HPS3, HPS5, and HPS6 subunits, which can interact with BLOC‐1 and that localize to recycling endosomal tubulovesicular structures proximal to pigmented melanosomes (Di Pietro et al. 2006) (Figure 1b). Mutations in BLOC‐2 subunits typically result in mild hypopigmentation and hemorrhagic diathesis, though some cases of granulomatous colitis were also reported (Huizing et al. 2020) (Table 1).

Functioning downstream of BLOC‐1, BLOC‐2 plays a critical role in directing endosomal‐derived tubules to LROs. In pigment cells, its loss does not prevent the formation of BLOC‐1‐dependent recycling tubules, but affects their dynamics by reducing the number and duration of their contacts with pigmented melanosomes (Dennis et al. 2015). These process might rely on RAB22A, as this GTPase interacts with BLOC‐2 and promotes its membrane association (Shakya et al. 2018).

In endothelial cells, BLOC‐2 directs endosomal‐derived tubules to their LRO, the Weibel–Palade body (WPB) (Sharda et al. 2020), further supporting its function as a general endosomal tether connecting BLOC‐1‐dependent tubules to LROs. Recently, an ancestral four‐subunit BLOC‐2 complex has been identified in the unicellular eukaryote *Dictyostelium*, where it localizes to endolysosomes and regulates their maturation by associating with the WASH complex (Thomason et al. 2024). WASH, in its turn, promotes local branched actin polymerization for membrane remodeling at organelles like early endosomes (Seaman et al. 2013). Consequently, in mammalian systems, BLOC‐2 may perhaps associate with the limiting membranes of both recycling endosomal tubules and LROs, either acting *in trans* as a molecular tether to stabilize membrane contacts or cooperating with other tethering complexes, such as the exocyst that interacts with BLOC‐2 and promotes recycling endosome contacts with plasma membrane or WBPs (Sharda et al. 2020). Altogether, and although its exact mechanism of action remains to be elucidated, BLOC‐2 emerges as a regulator of endosomal tubule dynamics and their trafficking to target membranes—such as LROs or the plasma membrane—thus potentially contributing to processes like LRO maturation or cargo recycling, respectively.

### 2.3 From Endosomal Subdomain Organization to Cargo Selection and Trafficking: The Role of AP‐3

At early endosomes, cargo destined for LROs must be selectively sorted into newly formed tubulovesicular carriers, a process regulated in part by heterotetrameric adaptor complexes such as AP‐1 and AP‐3 in melanocytes (Delevoye et al. 2009; Setty et al. 2008; Sitaram et al. 2012, 2009; Theos et al. 2005). In HPS, mutations in AP3B1 or AP3D1, encoding the β3A and δ subunits of AP‐3, cause HPS2 and HPS10, respectively, leading to severe immunodeficiency, pulmonary fibrosis, and neurological symptoms, particularly in HPS10 (Huizing et al. 2020; Introne et al. 1993) (Table 1). In pigment cells, AP‐3 recognizes melanosomal cargos like TYR through dileucine‐based sorting signals (Bonifacino and Traub 2003) and promotes its selection and packaging into vesicles budding from early endosomes and transported to melanosomes (Theos et al. 2005) (Figure 1a). AP‐1 can also bind to the dileucine‐based sorting signal of TYR, and a small fraction of TYR co‐distributes with AP1 clathrin–coated structures in mouse melanocytes (Theos et al. 2005). This fraction increases dramatically in AP‐3‐deficient mouse melanocytes, proposing together that AP‐1 may partially compensate for AP‐3 deficiency through rescue of the TYR trafficking to melanosomes via the AP‐1/BLOC‐1 tubular route (Bowman et al. 2021; Delevoye et al. 2009; Theos et al. 2005). This functional overlap highlights the existence of interconnected, redundant yet distinct intracellular pathways, which may provide robustness to the endosome–melanosome trafficking route, as also reflected during endosomal sorting of the SNARE VAMP7 to melanosomes (see above) via the AP‐3/BLOC‐1 “super‐complex” (Bowman et al. 2021). Beyond cargo selection, AP‐3 also contributes to organizing membrane subdomains at early endosomes, optimizing cargo sorting and vesicular transport (Nag et al. 2018). Therefore, AP‐3 plays a broader role in endosomal domain specialization, helping to define distinct trafficking routes that ultimately converge on the same target organelles.

### 2.4 Membrane Tubulation From LRO: A Recycling Function Driven by BLOC‐3?

Organelle maturation is essential for establishing and maintaining their morphological, molecular, and functional identities, a process largely driven by membrane dynamics. Endolysosomal organelles can be viewed as metastable structures—appearing stable while continuously balancing the exchange of material exchange through import/export trafficking pathways. In this context, BLOC‐3, a complex composed of HPS1 and HPS4 (Nazarian et al. 2003), plays a crucial role in coordinating this process at the melanosome level (Figure 1c).

Mutations in HPS1 or HPS4 genes lead to severe forms of OCA, hemorrhagic diathesis, granulomatous colitis, and pulmonary fibrosis characterized by giant lamellar bodies in alveolar epithelial cells, often proving fatal in midlife (Huizing et al. 2020) (Table 1). Functioning as a guanine nucleotide exchange factor (GEF), BLOC‐3 activates RAB32 and RAB38 (Gerondopoulos et al. 2012), which play overlapping roles in cells generating LROs, such as melanosomes, dense granules, and lamellar bodies (Bowman et al. 2019). In pigment cells, active RAB32/38 associate with the limiting membrane of pigmented melanosomes (Wasmeier et al. 2006), with RAB38 also localizing to dynamic tubules released from melanosomes—structures absent in HPS4‐deficient cells (Dennis et al. 2016). Thus, BLOC‐3 appears to promote the formation of membrane tubules from pigmented melanosomes (Figure 1), acting downstream of RAB32/38 membrane association.

Given that melanosomes are still pigmented in the absence of BLOC‐3, why do patients develop albinism? While initial studies suggested BLOC‐3 aids in the delivery of melanogenic enzymes to melanosomes [for more details, see Bowman et al. 2019], recent findings implicate BLOC‐3 in membrane remodeling and tubule release from pigmented melanosomes (Figure 1c). Overall, membrane tubules have a distinctive geometry, with a high membrane‐to‐lumen ratio, which may facilitate their enrichment with lipids and membrane‐associated proteins, akin to endosomal tubules involved in receptor sorting and recycling (Maxfield and McGraw 2004). BLOC‐3‐dependent melanosomal tubules are significantly wider than BLOC‐1‐dependent recycling endosomal tubules [∼150 nm vs. 60 nm in diameter, respectively; Jani et al. 2022; Ripoll et al. 2018], potentially facilitating the concentration of luminal solutes in addition to membrane‐associated components. Disrupting actin‐dependent melanosomal tubule release leads to enlarged, highly melanized melanosomes with increased acidification, which is associated with defective pigment secretion and transfer to keratinocytes (Loubery et al. 2012; Ripoll et al. 2018). Similarly, in Type II alveolar epithelial cells of BLOC‐3‐deficient mice, lamellar bodies were characterized as enlarged and poorly secretory (Guttentag et al. 2005), which could underlie a cellular process contributing to progressive lung fibrosis in humans. Intriguingly, lysosomal exocytosis in *Dictyostelium* also relies on actin‐dependent membrane tubulation and pH alkalinization through V‐ATPase removal (Carnell et al. 2011). Thus, although still speculative, BLOC‐3 may orchestrate RAB32/38 recruitment to LROs or secretory lysosomes to drive large membrane tubule biogenesis and release, crucial for luminal homeostasis and organelle exocytosis. Altogether, the OCA phenotype observed in HPS1 or HPS4 patients (Table 1) may result from abnormal pigmented melanosomes with impaired capacity to secrete or transfer pigment, ultimately causing hypopigmentation at the skin level.

The final destination of BLOC‐3‐dependent melanosomal tubules remains unclear. One hypothesis suggests that these tubules may return back to early endosomes/Stage I melanosomes (Figure 1), recycling SNAREs like VAMP7 to support further membrane fusion events between BLOC‐1‐dependent recycling endosomal tubules and maturing melanosomes (Dennis et al. 2016). If true, this model predicts that defective melanosomal recycling could reduce available trafficking components, thereby indirectly impairing melanosome biogenesis and ultimately reducing pigment production.

## 3 The Keratinocyte—the Pigment Warehouse

**FIGURE 3:**
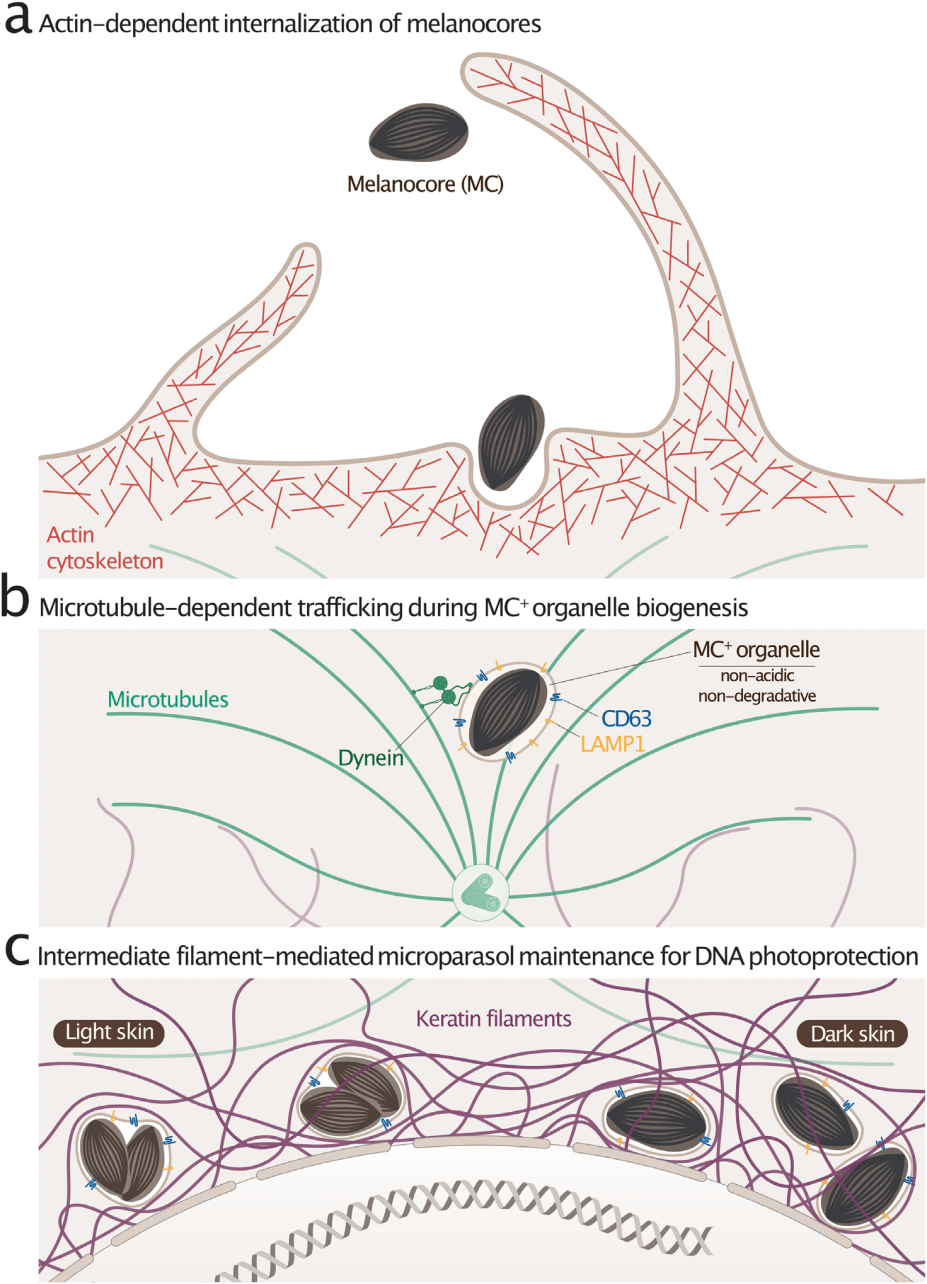
Contribution of the keratinocyte cytoskeleton to melanocore internalization, melanocore‐containing organelle biogenesis, and trafficking. The coordinated action of the actin, microtubule, and intermediate filament cytoskeletal systems ensures the strategic spatial distribution of melanin within keratinocytes, enabling effective protection of nuclear DNA from ultraviolet‐induced damage. (a) Secreted melanin particles, or melanocores (MCs), are internalized by keratinocytes through an actin‐dependent process, likely involving the Rho GTPases Rac1 and Cdc42. (b) The resulting MC‐containing organelles (MC^+^ organelles) follow a conventional endosomal pathway, ultimately acquiring late endosomal/lysosomal markers such as LAMP1 and CD63, albeit nonacidic and nondegradative. MC^+^ organelles are trafficked retrogradely toward the perinuclear region via dynein motor proteins along microtubules, promoting their accumulation near the nucleus. (c) Intermediate filaments composed of keratin‐5 and keratin‐14 help maintain the perinuclear and apical positioning of MC^+^ organelles by forming cytoskeletal cages that retain them above the nucleus, ensuring their photoprotective function.

Melanin pigments produced by melanocytes are transferred to keratinocytes, the predominant epidermal cells. Like an optimized warehouse, keratinocytes ensure long‐term storage and efficient distribution of melanin throughout the epidermis. Within keratinocytes, transferred melanin is newly repackaged into de novo pigment organelles resembling LROs and melanosomes, which contribute to skin color and provide protection against UV solar radiation (Figure 3). While most insights into membrane dynamics in pigmentation come from melanocytes, similar trafficking events likely occur in keratinocytes. Although evidence remains limited, emerging data suggest that the keratinocyte endolysosomal system plays a central role in melanin uptake, organelle biogenesis, maturation, positioning, and photoprotective function.

### 3.1 Internalization of Pigments

Several mechanisms have been proposed for melanin transfer from melanocytes to keratinocytes (see Benito‐Martínez et al. 2021). Most transfer models, such as cytophagocytosis, shedding vesicles, or the tunneling nanotubes models, suggest that melanin is transferred as an intact melanosome, which might be surrounded by an additional keratinocyte‐derived membrane once internalized. However, in situ analysis of human skin keratinocytes consistently shows pigment organelles with a single limiting membrane that lacks melanosomal components (Benito‐Martínez et al. 2025; Hall et al. 2022; Hurbain et al. 2018; Tarafder et al. 2014). This observation strongly supports the “coupled exocytosis/endocytosis” transfer model (Figure 3a), which is currently supported by the majority of in vitro data and analysis of human skin. According to this, melanocytes release the intraluminal melanin core (i.e., melanocore, MC) contained in the melanosome into the extracellular space through its fusion with the plasma membrane. Keratinocytes then internalize MCs, enclosing them in a single membrane of keratinocyte origin (Benito‐Martínez et al. 2025; Correia et al. 2018; Hall et al. 2022; Hurbain et al. 2018; Tarafder et al. 2014) (Figure 3a,b).

Despite mechanistic differences, all melanin transfer models require plasma membrane remodeling through the actin cytoskeleton to internalize these large melanin particles (∼0.5 µm; Benito‐Martínez et al. 2025). Studies suggest that melanin uptake involves Rho GTPases (Koike et al. 2019; Moreiras et al. 2022; Scott et al. 2002) and that actin depolymerization, via cytochalasin D or latrunculin A, inhibits keratinocyte phagocytosis of MCs (Moreiras et al. 2022). Recent findings further indicate that MC internalization relies on Rac1‐ or Cdc42‐dependent phagocytosis, whereas melanosome uptake requires macropinocytosis and RhoA activity (Moreiras et al. 2022). While these data suggest that keratinocytes may engage distinct recognition, capture and internalization pathways depending on the nature of the melanin particles, complementary or overlapping mechanisms cannot be ruled out. Finally, whether MC‐associated components contribute to keratinocyte recognition and uptake remains an open question.

Components associated with MCs may activate the protease‐activated receptor‐2 (PAR‐2), which is linked to melanin internalization but lacks specificity as it also facilitates the uptake of melanosomes, synthetic melanin, beads, or bacterial bioparticles (Moreiras et al. 2022; Scott et al. 2003; Seiberg et al. 2000; Sharlow et al. 2000). In addition, the predominant expression of PAR‐2 in the upper epidermal granular layer (Fan et al. 2024; Steinhoff et al. 1999) suggests a limited role in direct melanin transfer between melanocytes and keratinocytes in the basal layer. PAR‐2 activity likely enhances keratinocyte phagocytic capacity via actin remodeling and Rho GTPase activation (Greenberg et al. 2003; Scott et al. 2003; Sharlow et al. 2000). Though its mechanism remains unclear, PAR‐2 activation by proteases also leads to intracellular calcium changes, potentially aiding particle uptake as well as triggering pro‐inflammatory responses (Fan et al. 2024). Notably, PAR‐2 activity is strongly linked to skin inflammation and barrier function in conditions like acne vulgaris, rosacea, psoriasis, atopic dermatitis, or systemic sclerosis (Fan et al. 2024).

### 3.2 Biogenesis of the Pigment Organelle in Keratinocytes

Within keratinocytes, MCs reside in a unique organelle variably named in the literature, including *melanokerasomes* or *MC‐positive (MC^+^) organelles*, and sometimes misleadingly referred to as *melanosomes*. Here, we use the term “MC^+^ organelle” to explicitly describe the pigment‐containing organelle of keratinocytes (Figure 3). In both human skin and in vitro models, MC^+^ organelles exhibit late endosomal/lysosomal features, bearing LAMP1 and CD63 proteins (Benito‐Martínez et al. 2025; Correia et al. 2018; Hurbain et al. 2018; Neto et al. 2025) (Figure 3b). However, unlike conventional lysosomes, MC^+^ organelles show low acidity and degradative capacity (Benito‐Martínez et al. 2025; Correia et al. 2018; Hurbain et al. 2018) (Figure 3b). Although their biogenesis remains unclear, evidence suggests that within 24 h of MC uptake, MC^+^ organelles follow a classical endosomal pathway, initially acquiring early endosome markers (EEA1, RAB5) and then late endosome components (CD63, LAMP1, LAMP2) while fusing with conventional lysosomes (Correia et al. 2018; Neto et al. 2025). Like melanosomes and some LROs, MC^+^ organelles likely begin as acidic, degradative structures before maturing into specialized, nonacidic, nondegradative compartments (Figure 3b).

The maturation of MC^+^ organelles suggests that, similarly to melanosomes, they undergo membrane remodeling events that allow for progressive identity changes through the import/export of material. This concept is supported by data revealing that pigmented melanosomes are internalized, rather than MCs, for 2 days by mouse keratinocytes co‐distribute with no fewer than 11 RAB GTPases (i.e., Rab7B/42, 19, 25, 27A/B, 32, 33A, 37, 38, 39A, and 44) (Marubashi and Fukuda 2020). While the role of most of these RABs remains unclear, RAB7 would be required for the initial fusion of pigment organelles with lysosomes, and hence their first degradative phase, regardless of melanosome or MC uptake (Marubashi and Fukuda 2020; Neto et al. 2025). Despite this early degradative identity, melanin persists in keratinocytes for several days, both in situ and in vitro (Benito‐Martínez et al. 2025; Correia et al. 2018; Hurbain et al. 2018; Marubashi and Fukuda 2020). Attempts to hydrolytically degrade melanin in vitro led to protein breakdown, yet the melanin moiety remained intact (Ito and Wakamatsu 1998; Otaki and Seiji 1971). Similarly, melanosome uptake by mouse keratinocytes results in degradation of melanosomal proteins (e.g., TYRP1), while the melanin content in the cells is maintained (Marubashi and Fukuda 2020). Consistent with these findings, electron microscopy studies across human skin, reconstructed epidermis, and keratinocytes fed with MC consistently show MC^+^ organelles containing intact MCs (Benito‐Martínez et al. 2025; Correia et al. 2018; Hurbain et al. 2018; Neto et al. 2025; Tarafder et al. 2014). Interestingly, treatment of keratinocytes or ex vivo skin biopsies with autophagy modulators leads to altered tissue pigmentation, suggesting that autophagy might influence pigment dynamics (Kim et al. 2020; Murase et al. 2013). While melanin degradation in keratinocytes remains debated, MC^+^ organelles likely function as specialized endolysosomal compartments for long‐term melanin storage, regardless of skin color type.

The organization and content of pigment organelles in keratinocytes vary with skin color. In light to moderately pigmented skin, multiple MCs cluster within a single‐membrane organelle in basal keratinocytes. In contrast, darker skin displays individual membrane‐bound MCs dispersed throughout both basal and suprabasal layers (Hurbain et al. 2018; Minwalla et al. 2001; Szabó et al. 1969; Thong et al. 2003; Yoshida et al. 2007). Whether these differences result from organelle fusion–fission events during maturation, or arise during MC uptake, remains unknown. Future studies should aim to elucidate the mechanisms of MC^+^ organelle biogenesis, membrane remodeling, trafficking mechanisms, and potential defects in disease.

### 3.3 Cytoskeleton‐Driven Pigment Organelle Spatialization for Genome Photoprotection

Once formed, MC^+^ organelles strategically localize perinuclearly and atop the nucleus of keratinocyte, forming a supranuclear melanin cap or microparasol (Gibbs et al. 2000; Kobayashi et al. 1998) (Figure 3c). This 3D organization concentrates photoprotective pigments, thus shielding DNA from solar UV‐induced damage (Brenner and Hearing 2008; Del Bino et al. 2006). However, the mechanisms governing microparasol formation and maintenance, as well as their potential alteration in disease, are still poorly understood.

During biogenesis and maturation, MC^+^ organelles undergo retrograde transport from the cell periphery to the perinuclear area (Neto et al. 2025). This long‐distance centripetal motion classically relies on microtubules, the minus‐end directed motor dynein and its adaptor p150^Glued^, which localize at the supranuclear melanin cap (Byers et al. 2007, 2003) (Figure 3b). Disrupting microtubule polymerization or F‐actin dynamics alters the vertical positioning of the melanin cap in ex vivo human skin samples and in keratinocyte models fed with MCs (Benito‐Martínez et al. 2025; Castellano‐Pellicena et al. 2021). The polarization of MC^+^ organelles atop the nucleus aligns with centrosome orientation and its associated machineries (Castellano‐Pellicena et al. 2021), and depends on plectin (PLEC) (Benito‐Martínez et al. 2025)—a cytolinker bridging microtubules to intermediate filaments (Castañón et al. 2013). These findings suggest that keratinocytes are polarized cells exploiting cytoskeletal networks to position MC^+^ organelles in close proximity to their nucleus.

Beyond their vertical positioning, the 3D‐spatialization of MC^+^ organelles requires a third cytoskeletal network—the intermediate filaments composed of keratin‐5 and ‐14 (KRT5/14) (Benito‐Martínez et al. 2025) (Figure 3c). These filaments form cytoskeletal cages that locally rigidify the cytosol and trap MC^+^ organelles near and atop the nucleus, thereby contributing to genome photoprotection (Benito‐Martínez et al. 2025). Diseases associated with KRT5 or KRT14 haploinsufficiency, such as Dowling–Degos disease (DDD; KRT5), epidermolysis bullosa simplex (KRT5, KRT14), Naegeli‐Franceschetti‐Jadassohn syndrome, or dermatopathia pigmentosa reticularis (KRT14), are in part characterized by hyperpigmentation (Table 2). Importantly, hyperpigmented skin lesions in body folds of DDD patients are also associated with a sparse intracellular pigment distribution in basal keratinocytes (Betz et al. 2006) (Table 2). Such subcellular dispersion of pigment organelles is recapitulated in a human keratinocyte model carrying the KRT5^DDD^ mutation and fed with MCs (Benito‐Martínez et al. 2025). Thus, keratinocytes coordinate their three cytoskeletal systems—actin, microtubules, and intermediate filaments—to mediate MC uptake and strategically maintain MC^+^ organelles around the nucleus as an effective UV‐protective mechanism (Figure 3). Finally, for a given number of MC^+^ organelles, their physical proximity to the nucleus is a key factor in genome photoprotection (Benito‐Martínez et al. 2025). Thus, such patients suffering from diseases associated with hyperpigmentation and pigment organelle dispersion in keratinocytes may counterintuitively display an increased susceptibility to develop skin cancer due to suboptimal photoprotection.

**TABLE 2:** Keratinocyte pigmentation disorders with potential defects in membrane dynamics processes.

Gene	Protein name	Function	Human disease and clinical manifestation
*KRT5*	Keratin, type II cytoskeletal 5	Type II cytokeratin is specifically expressed in the basal layer of the epidermis, which pairs with KRT14 to form keratin intermediate filaments that facilitate the 3D‐positioning of MC^+^ organelles in vitro and in vivo.	**Epidermolysis bullosa simplex** Skin blistering and fragility Nail dystrophy Mottled/Reticulate hyperpigmentation Focal hyperkeratosis **Dowling–Degos syndrome** Reticulate hyperpigmentation Hyperkeratotic papules
*KRT14*	Keratin, type I cytoskeletal 14	Type I cytokeratin is specifically expressed in the basal layer of the epidermis, which pairs with KRT5 to form keratin intermediate filaments that facilitate the 3D‐positioning of MC^+^ organelles in vitro.	**Epidermolysis bullosa simplex** Skin blistering and fragility Nail dystrophy **Naegeli–Franceschetti–Jadassohn syndrome** Reticular hyperpigmentation Absence of fingerprints Hypohidrosis Nail dystrophy Tooth enamel defect **Dermatopathia pigmentosa reticularis** Reticular hyperpigmentation Alopecia Nail dystrophy

## 4 Conclusions and Perspectives

Through its ability to adapt to environmental cues and respond to cellular demands, the endolysosomal system demonstrates remarkable plasticity. This adaptability is exemplified by the diversity of membrane remodeling processes that drive the formation of intracellular carriers, which are essential not only for cargo delivery but also for maintaining organelle homeostasis and function. Disruption of these dynamic processes can severely compromise the functional and adaptive capacities of cells and tissues.

Specialized cells, such as those that synthesize, uptake, and store melanin, illustrate how the endolysosomal system and its membrane dynamics can be harnessed to meet unique physiological demands. Through the generation of de novo organelles and the orchestration of protective responses against daily genotoxic stressors like solar UV radiation, these cells underscore the broader relevance of membrane trafficking in cellular defense and adaptation. Perturbations in these trafficking pathways and membrane remodeling processes are increasingly recognized to underlie various pathological conditions, including genetic disorders.

To date, syndromic albinism—such as HPS—is clinically defined as a melanocyte‐related disorder, and patients with this condition exhibit wide variability in skin pigmentation. While the role of melanocytes in melanin and melanosome synthesis is well established, the contribution of keratinocytes to pigmentation and photoprotection in these patients remains unexplored. This is particularly striking given that BLOCs and AP‐3 complexes are ubiquitously expressed, making it highly plausible that these complexes—and the trafficking steps they regulate—may also be involved in keratinocyte biology, contributing to processes such as pigment internalization and/or the formation, maturation, and positioning of the resulting pigmented and photoprotective organelles. Uncovering the putative involvement of BLOCs and/or AP‐3 in keratinocyte biology would not only expand the functional landscape of these complexes but also position MC^+^ organelles within the LRO family, thus marking a step forward in our understanding of skin pigmentation, photoprotection, and the pathophysiology, diagnosis, and clinical management of syndromic forms of albinism. For instance, the clinical manifestations of DDD demonstrate that keratinocytes contribute to skin pigmentation. Therefore, a more integrated view of the mechanisms underlying the pigmentary manifestations of certain diseases can be achieved by understanding the biology of MC and its organelles in keratinocyte, ultimately providing deeper insight into the interplay between melanocytes and keratinocytes.

Altogether, the study of membrane trafficking and dynamics in the skin stands as a compelling example—if one was still needed—of how fundamental cell biology can shed light on the etiology of diseases.

## Author Contributions

Conceptualization: Laura Salavessa and Cédric Delevoye. Investigation: Laura Salavessa, Myckaëla Rouabah, Paula Pernea, Smail Hadj‐Rabia, and Cédric Delevoye. Writing original draft preparation and editing: Laura Salavessa, Myckaëla Rouabah, Paula Pernea, Smail Hadj‐Rabia, and Cédric Delevoye. Visualization: Laura Salavessa and Cédric Delevoye. Supervision and project administration: Cédric Delevoye. Funding acquisition: Smail Hadj‐Rabia and Cédric Delevoye.

## Conflicts of Interest

The authors declare no conflicts of interest.
